# Volume and accumulation patterns of physical activity and sedentary time: longitudinal changes and tracking from early to late childhood

**DOI:** 10.1186/s12966-021-01105-y

**Published:** 2021-03-17

**Authors:** Katherine L Downing, Trina Hinkley, Anna Timperio, Jo Salmon, Alison Carver, Dylan P Cliff, Anthony D Okely, Kylie D Hesketh

**Affiliations:** 1 Institute for Physical Activity and Nutrition (IPAN), School of Exercise and Nutrition Sciences, Deakin University, Geelong, Australia; 2 Mary MacKillop Institute for Health Research, Australian Catholic University, Melbourne, Australia; 3 Early Start, Faculty of Social Sciences, Illawarra Health and Medical Research Institute, University of Wollongong, Wollongong, Australia

**Keywords:** Sedentary behaviour, Physical activity, Stability, Longitudinal, Preschool, School‐aged

## Abstract

**Background:**

Physical activity (PA) decreases and sedentary time (SED) increases across childhood, with both behaviours tracking. However, no studies have examined how accumulation patterns of PA and SED (i.e., prolonged bouts, frequency of breaks in sedentary time) change and track over time. The aim of this study was to investigate longitudinal changes in and tracking of total volume and accumulation patterns of SED, light-intensity PA (LPA), moderate-intensity PA (MPA), vigorous-intensity PA (VPA) among boys and girls.

**Methods:**

In 2008/09 (T1), children in HAPPY (3-5y; *n* = 758) in Melbourne, Australia wore ActiGraph GT1M accelerometers to objectively assess SED, LPA, MPA and VPA. This was repeated at age 6-8y (T2; *n* = 473) and 9-11y (T3; *n* = 478). Ten pattern variables were computed: bouts of ≥ 5-, ≥ 10-, ≥ 15- and ≥ 20-min for SED, ≥ 1- and ≥ 5-min for LPA, ≥ 1-min for MPA, ≥ 1- and ≥ 5-min for VPA, and breaks in SED (interruptions of > 25 counts 15 s^− 1^). Longitudinal mixed models examined changes from T1-3, controlling for T1 age. Generalized estimating equations assessed tracking over the three time points, controlling for T1 age and time between measurements. Analyses were stratified by sex.

**Results:**

Total volume and bouts of SED and SED breaks increased, while total volume and bouts of LPA decreased for both sexes. There was a small decrease in total volume of MPA for girls, but time spent in ≥ 1-min bouts increased for both sexes. Total volume of VPA increased for both sexes, with time spent in ≥ 1-min bouts increasing for boys only. All volume and pattern variables tracked moderately for boys, except for all SED bouts ≥ 15-min, LPA bouts ≥ 5-min and MPA bouts ≥ 1-min (which tracked weakly). For girls, total SED and SED bouts ≥ 1-min tracked strongly, total volume of LPA, MPA and VPA, ≥ 5- and ≥ 10-min SED bouts, and ≥ 1-min LPA and MPA bouts tracked moderately, and SED breaks, all SED bouts ≥ 15 min, LPA bouts ≥ 5 min and all VPA bouts tracked weakly.

**Conclusions:**

Patterns of SED and PA change from early to late childhood; with the exception of SED breaks and VPA, changes were detrimental. Total volumes and short bouts tended to track more strongly than longer bouts. Interventions to prevent declines in PA and increases in SED are important from early in life.

## Introduction

Engaging in sufficient physical activity and minimising time spent sedentary contribute to healthy development during and beyond early childhood [1, 2]. In addition to total volume, accumulation patterns of physical activity and sedentary time (i.e., the number and duration of bouts and breaks in sedentary time) may be important for children’s health. Although the benefits of reducing sedentary bouts are yet to be established in young children, breaking up prolonged bouts of sedentary time has been shown to be associated with more favourable cardio-metabolic profiles in school-aged children [3–5].

Although Australian preschool children (aged 3- to 5-years) spend around 5–6 h per day being physically active [6, 7], with the majority meeting the physical activity guidelines of 180 min/day [6, 8], their physical activity is sporadic in nature [7]. In addition, they spend more than 50 % of their waking hours sedentary [7]. Further, international cross-sectional data (n > 27,000) indicate that from age 5 years, when preschool children begin their transition to primary (elementary) school, physical activity starts to decline rapidly and sedentary time starts to increase, with boys more active and less sedentary than girls at all ages [9].

In addition to determining the nature of changes in physical activity and sedentary time, it is important to examine how these behaviours track. “Tracking” refers to the stability of a particular behaviour over time [10], or the maintenance of an individuals’ relative rank over time within a cohort [11]. Jones et al. [12] conducted a systematic review of studies reporting tracking of physical activity and sedentary behaviours from early childhood. That review found evidence of moderate (coefficient 0.30–0.49) tracking of physical activity, and moderate to strong (coefficient ≥ 0.50) tracking of sedentary behaviour (mostly in the form of sedentary screen time), during early childhood and from early childhood to middle childhood. However, just two of the 14 studies included in that review reported tracking of objectively assessed sedentary time, and six of the 14 studies reported tracking of objectively-assessed physical activity.

Studies published since that review that used accelerometers have reported similar results. Carson et al. [13] found moderate tracking for light-intensity physical activity (LPA), MVPA and sedentary time over three time points during early childhood (from age 1.6 to 3.7 years). Caldwell et al. [14] found moderate tracking for LPA, moderate-intensity physical activity (MPA) and vigorous-intensity physical activity (VPA), as well as strong tracking for sedentary time over 1 year (age 4.5 to 5.5 years). Carson et al. [15] investigated tracking of sedentary time and bouts over a 1 year period among 3- to 5-year-olds, and found moderate tracking of total sedentary time, 1–4 min sedentary bouts, and 5–9 min sedentary bouts, but no tracking of ≥ 10 min sedentary bouts. No studies have investigated tracking in boys and girls separately and no studies have investigated tracking of total volumes and accumulation patterns (bouts) of objectively measured physical activity and sedentary time over multiple time points. Identifying whether specific patterns of accumulation of sedentary time and physical activity track may help inform future intervention strategies. For example, if children’s engagement in long bouts of sedentary time track moderately to strongly, future interventions could include approaches to break up prolonged sitting from a young age. The aim of this study was to investigate longitudinal changes and tracking of total volumes and patterns of accumulation of sedentary time, LPA, MPA and VPA from preschool (3–5 years) to late primary school (9–11 years) among boys and girls.

## Methods

### Recruitment and participants

Data for this study were from the Healthy Active Preschool and Primary Years (HAPPY) cohort study. Recruitment details for this study have been previously published [16]. Briefly, parents and their children were recruited from preschools and childcare centres in August-December 2008 and June-November 2009 in Melbourne, Australia. All parents with children aged 3–5 years attending participating centres were invited to participate, with 1002 parents/guardians providing consent at baseline (T1).

Although initially designed as a cross-sectional study, 766 parents (77 %) provided consent to be re-contacted for future research. These families were invited to participate in follow-up studies at three years post-baseline (from August 2011-March 2012 and June 2012-April 2013; T2) and at six years post-baseline (from July 2014-May 2015 and June 2015-May 2016; T3). A total of 567 (74 %) and 568 (76 %) parents consented to T2 and T3, respectively. Deakin University Human Research Ethics Committee (EC291-2007), Department of Education and Early Childhood Development (2011_001008), and Catholic Education Melbourne (GE11/0009) approved the study. Parents provided written informed consent and children provided verbal assent to wear the accelerometers at each time point.

### Measures and data management

At T1, parents reported their child’s sex and date of birth (used to calculate age). Children were visited at preschool at T1, and at T2 and T3 parents opted for their child to be visited either at home or school, with data collected during school terms. Children were fitted with an ActiGraph GT1M uniaxial accelerometer (Pensacola, FL, USA) on an elastic belt at the right iliac crest. They were instructed to wear it during waking hours for eight consecutive days, only removing for water-based activities (e.g., bathing, swimming). Data were collected in 15-second epochs [17, 18] and were processed using a customised Microsoft Excel macro. Non-wear time was determined as ≥ 20 min of consecutive zero counts [17, 19]. Children were required to have data recorded for ≥ 6 h per day (at T1) and ≥ 8 h per day (at T2 and T3) on ≥ 4 days (including ≥ 1 weekend day) [20, 21] to be included in the analyses. The lower wear time criterion used at T1 compared to T2 and T3 is specific to preschool children and accounts for their longer sleep time and subsequent shorter wake time (i.e., less time available for accelerometer wear time), compared with school-aged children. Sedentary time (SED) and physical activity were defined using cut points established by Evenson et al. [22](SED ≤ 25, LPA 26–573, MPA 574–1002, and VPA ≥ 1003 counts 15 s^− 1^), which have been validated in preschool [23] and school-aged [24] children.

Patterns of accumulation were defined a-priori as bouts of ≥ 5-min, ≥ 10-min, ≥ 15-min and ≥ 20-min of SED and ≥ 1-min, ≥ 5-min and ≥ 10-min of LPA, MPA, and VPA; however, only bout lengths that were recorded by ≥ 25 % of the sample were included in analyses. Hence, bouts included in analyses were ≥ 5-min, ≥ 10-min, ≥ 15-min and ≥ 20-min SED, ≥ 1-min and ≥ 5-min LPA, ≥ 1-min MPA, and ≥ 1-min and ≥ 5-min VPA bouts. Previous research has found that allowing exceptions (i.e., interruptions in intensity) increases the accumulation of time in longer bouts [25]; hence, no exceptions were allowed in this study. In addition, interruptions in SED time were defined as exceeding 25 counts 15 s^− 1^ [26]. Mean values for all accelerometry variables were calculated over all valid days; for bouts, mean values were calculated including only children who participated in those bout lengths (i.e., if a child did not engage in a particular bout length it was treated as missing) to reduce potential tracking of zeroes. All accelerometer variables were standardised using the residuals obtained when regressing on wear time [27].

### Data analysis

All analyses were conducted using Stata/SE 15.0 (StataCorp, Texas, USA) and were stratified by sex of the child. Descriptive analyses were used to summarise the data at each time point. Linear mixed models were used to determine change in each of the accelerometry variables (total time and bouts in each intensity [SED, LPA, MPA, VPA] and SED breaks), controlling for child’s age at T1. Mixed-models fit a linear trend line for each child and do not require data at each time point, maximising the use of available data. Tracking of each of the accelerometry variables were examined using General Estimating Equations (GEE), which use all available data to calculate a single stability coefficient. As per previous tracking studies [28, 29], GEE were used to predict the value of each variable at time t from the corresponding value at time t-1, controlling for age at T1 and time between measurements. Tracking coefficients were standardised by applying the formula:

$${\upbeta }\text{s} = {\upbeta }\left(\frac{\text{s}\text{d}(\text{Y}\text{t}-1)}{\text{s}\text{d}\left(\text{Y}\text{t}\right)}\right)$$

where β_s_ is the standardised tracking coefficient, β is the non-standardised tracking coefficient, sd(Y_t_ – 1) and sd(Y_t_) are the standard deviations of the outcome variables at times t–1 and t, respectively [30]. Tracking coefficients < 0.3 were considered weak, 0.3–0.6 moderate, and > 0.6 strong [11].

## Results

**Fig. 1:**
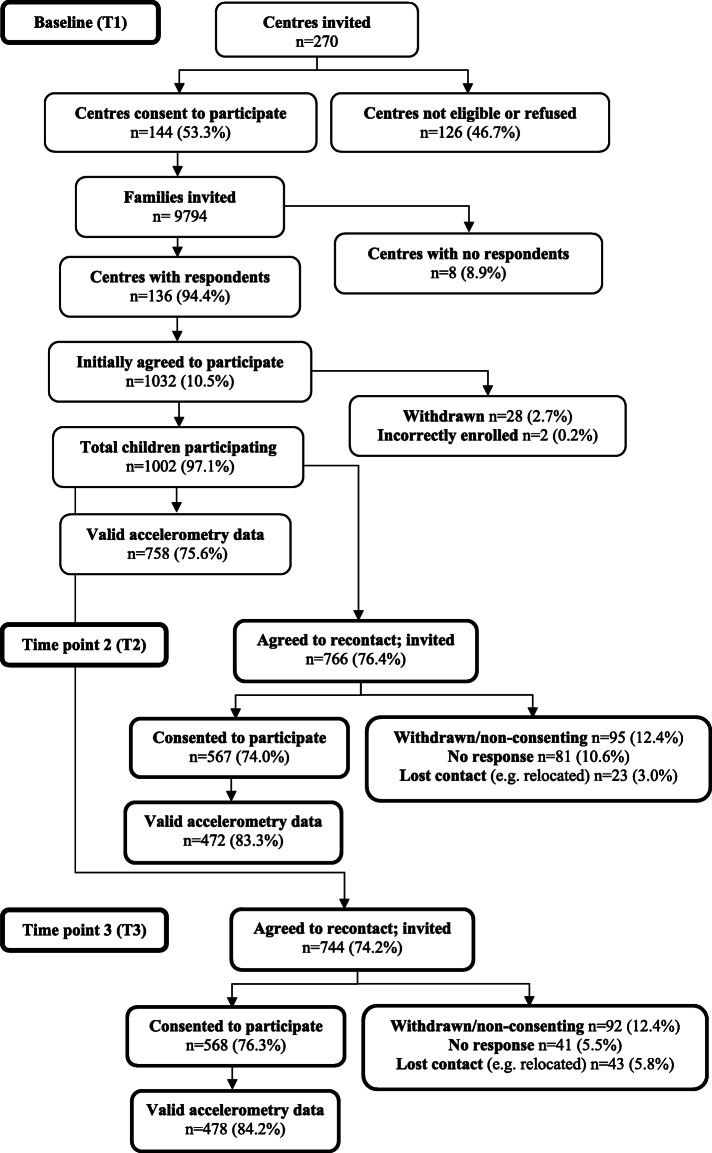
Participant flow chart

A total of 758 (76 %), 472 (83 %) and 478 (84 %) children had valid accelerometry data at T1, T2 and T3, respectively (see Fig. 1). There were no significant differences in baseline characteristics (child age, child sex, child BMI, maternal age, and maternal education) between those who participated at either follow up and those who did not (data not shown). Mean accelerometer wear times were 11.5 (SD = 1.2), 12.0 (SD = 1.0) and 12.3 (SD = 1.3) hours per day at T1, T2 and T3, respectively. Children had a mean age of 4.6 years (SD = 0.7) at T1, 7.6 years (SD = 0.7) at T2, and 10.6 years (SD = 0.7) at T3. At baseline, just over half (54 %) the children were boys and 55 % of mothers were university educated.

### Changes in volume and accumulation patterns of physical activity and sedentary time

**Fig. 2:**
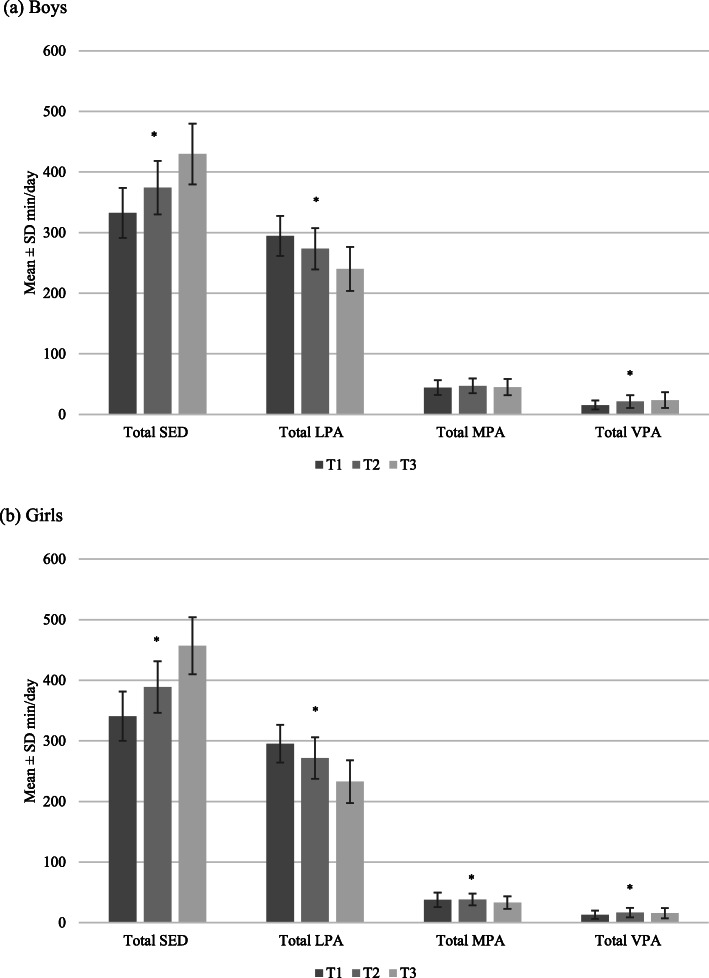
Total volumes of sedentary time and physical activity from T1 to T3 for boys and girls * *p* < 0.001 for change over time

**Fig. 3:**
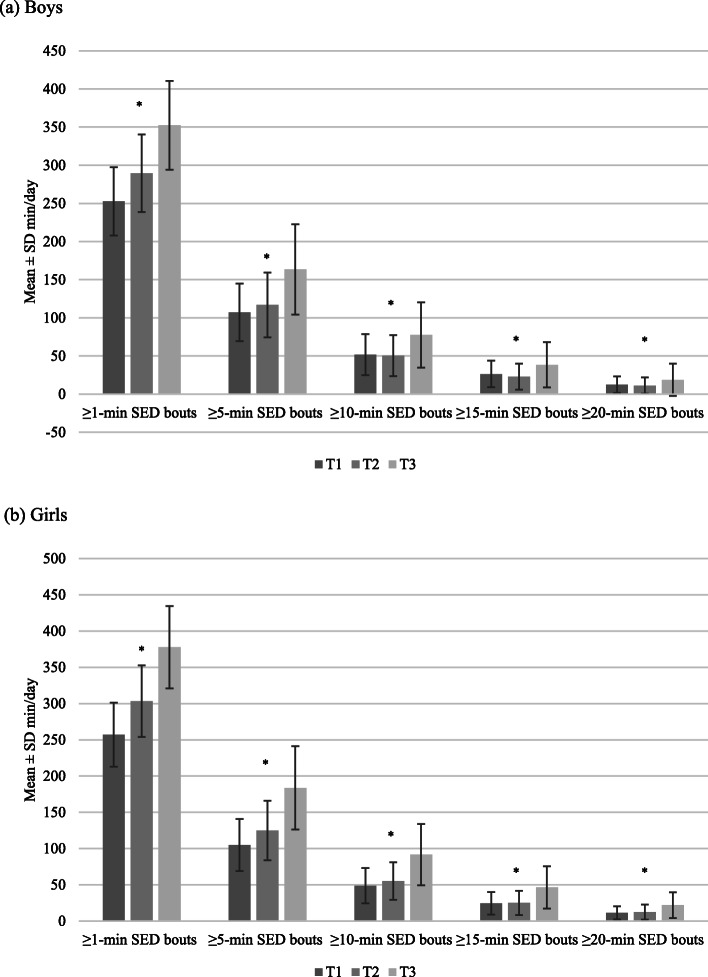
Bouts of sedentary time from T1 to T3 for boys and girls * *p* < 0.001 for change over time

**Fig. 4:**
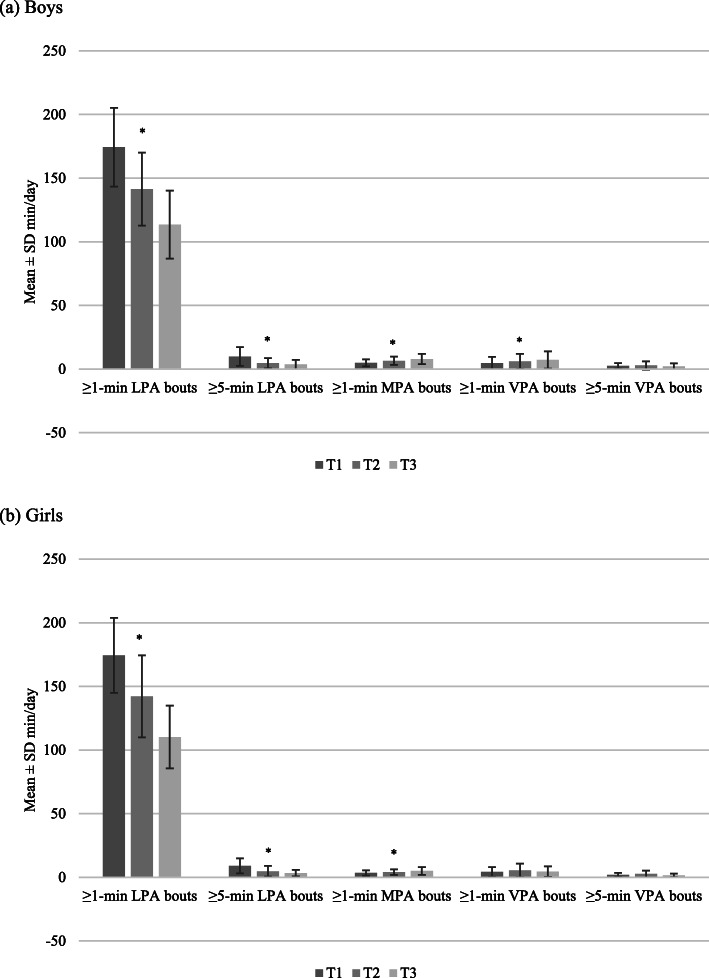
Bouts of physical activity intensities from T1 to T3 for boys and girls * *p* < 0.001 for change over time

**Fig. 5:**
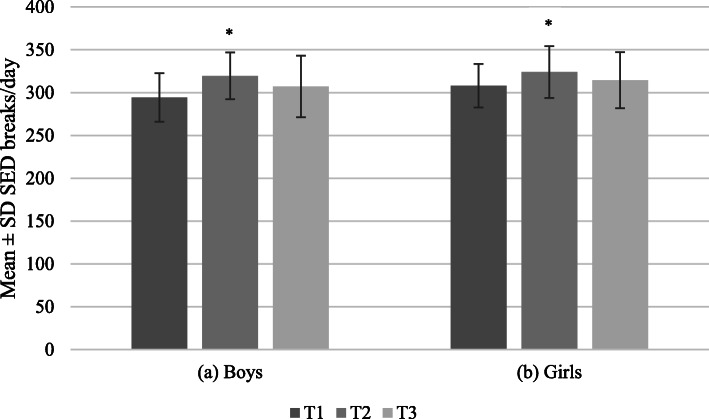
Breaks in sedentary time from T1 to T3 for boys and girls * *p* < 0.001 for change over time

Figures 2, 3 and 4 show the average total volumes of SED and physical activity intensities, bouts of SED, and bouts of physical activity intensities, respectively, for boys and girls. The average number of breaks in SED over time for boys and girls is shown in Fig. 5. At all timepoints, girls engaged in significantly more SED and less MPA and VPA than boys (all *p* < 0.001). There were no sex differences in average time spent in LPA until T3, where boys were more active than girls (*p* = 0.03). Coefficients for average change over time in each of the total volume and pattern variables are shown in Table 1. For both boys and girls, total volume of SED, breaks in SED, and all bout lengths of SED increased over time. Conversely, total volume and bouts of LPA decreased over time for both sexes. There was a small decrease in total volume of MPA for girls only; however, time spent in ≥ 1-min MPA bouts increased for both sexes over time. Total volume of VPA increased for both sexes, with time spent in ≥ 1-min bouts of VPA increasing for boys only, and no change observed for time spent in ≥ 5-min bouts for either boys or girls.

**Table 1:** Average change from T1 to T3 in total volume and accumulation patterns of physical activity and sedentary time

	β (95 % CI)^1^		
	Boys	Girls	Total sample
Total SED (min/day)	**48.2 (44.8, 51.7)**	**57.8 (54.1, 61.4)**	**52.7 (50.1, 55.2)**
Total LPA (min/day)	**-26.9 (-29.6, -24.3)**	**-31.2 (-34.0, -28.4)**	**-28.9 (-30.8, -27.0)**
Total MPA (min/day)	0.5 (-0.4, 1.5)	**-2.0 (-2.9, -1.1)**	-0.7 (-1.4, 0.1)
Total VPA (min/day)	**4.2 (3.4, 5.0)**	**1.4 (0.7, 2.0)**	**2.9 (2.4, 3.4)**
SED breaks (number/day)	**7.7 (5.2, 10.1)**	**4.2 (1.8, 6.6)**	**6.1 (4.3, 7.8)**
≥ 1-min SED bouts (min/day)	**49.0 (45.1, 52.9)**	**59.7 (55.5, 63.9)**	**54.0 (51.1, 56.9)**
≥ 5-min SED bouts (min/day)	**26.8 (23.2, 30.4)**	**38.2 (34.4, 42.0)**	**32.1 (29.5, 34.8)**
≥ 10-min SED bouts (min/day)	**12.0 (9.5, 14.5)**	**20.4 (17.8, 23.1)**	**15.9 (14.0, 17.7)**
≥ 15-min SED bouts (min/day)	**5.4 (3.7, 7.1)**	**10.3 (8.6, 12.1)**	**7.7 (6.4, 8.9)**
≥ 20-min SED bouts (min/day)	**2.9 (1.7, 4.2)**	**5.2 (4.0, 6.4)**	**4.0 (3.1, 4.8)**
≥ 1-min LPA bouts (min/day)	**-30.6 (-32.8, -28.4)**	**-32.6 (-34.9, -30.3)**	**-31.5 (-33.1, -29.9)**
≥ 5-min LPA bouts (min/day)	**-3.3 (-3.8, -2.9)**	**-3.1 (-3.5, -2.7)**	**-3.2 (-3.5, -2.9)**
≥ 1-min MPA bouts (min/day)	**1.5 (1.2, 1.7)**	**0.8 (0.6, 1.0)**	**1.2 (1.0, 1.4)**
≥ 1-min VPA bouts (min/day)	**1.3 (0.8, 1.7)**	0.2 (-0.2, 0.6)	**0.8 (0.5, 1.1)**
≥ 5-min VPA bouts (min/day)	-0.1 (-0.5, 0.2)	-0.1 (-0.4, 0.2)	-0.1 (-0.3, 0.2)

Notes: ^1^Mixed models controlling for age at T1; boldface indicates *p* < 0.05

Abbreviations: *CI* confidence interval; *LPA* light-intensity physical activity; *MPA* moderate-intensity physical activity; *SED* sedentary time; *VPA *vigorous intensity physical activity

### Tracking of volume and accumulation patterns of physical activity and sedentary time

Stability coefficients for each of the variables are shown in Table 2. For boys, there was moderate tracking of all volume and pattern variables except all SED bouts ≥ 15-min, LPA bouts ≥ 5-min and MPA bouts ≥ 1-min (which all tracked weakly). For girls, there was strong tracking for total SED and SED bouts ≥ 1-min. Moderate tracking was found for girls’ total volume of LPA, MPA and VPA, SED bouts ≥ 5-min and ≥ 10-min SED bouts, LPA bouts ≥ 1-min and MPA bouts ≥ 1-min, and weak tracking was found for girls’ SED breaks, all SED bouts ≥ 15-min, LPA bouts ≥ 5-min and all VPA bouts.

**Table 2:** Tracking coefficients for total volume and accumulation patterns of physical activity and sedentary time

	Standardised tracking (β) coefficients^1^		
	Boys	Girls	Total sample
Total SED (min/day)	**0.49**	**0.64**	**0.56**
Total LPA (min/day)	**0.52**	**0.58**	**0.54**
Total MPA (min/day)	**0.35**	**0.56**	**0.52**
Total VPA (min/day)	**0.54**	**0.60**	**0.58**
SED breaks (number/day)	**0.32**	**0.28**	**0.32**
≥ 1-min SED bouts (min/day)	**0.46**	**0.62**	**0.53**
≥ 5-min SED bouts (min/day)	**0.39**	**0.47**	**0.41**
≥ 10-min SED bouts (min/day)	**0.30**	**0.34**	**0.31**
≥ 15-min SED bouts (min/day)	**0.24**	**0.23**	**0.24**
≥ 20-min SED bouts (min/day)	**0.14**	**0.16**	**0.16**
≥ 1-min LPA bouts (min/day)	**0.60**	**0.42**	**0.53**
≥ 5-min LPA bouts (min/day)	**0.14**	0.03	0.05
≥ 1-min MPA bouts (min/day)	**0.22**	**0.33**	**0.35**
≥ 1-min VPA bouts (min/day)	**0.41**	**0.26**	**0.35**
≥ 5-min VPA bouts (min/day)	**0.40**	0.28	**0.43**

Notes: ^1^Tracking coefficients defined as weak (< 0.30), moderate (0.30–0.60), and strong (> 0.60); boldface indicates *p* < 0.05 for to non-standardised coefficients

Abbreviations: *LPA* light-intensity physical activity; *MPA* moderate-intensity physical activity; *SED* sedentary time; *VPA* vigorous intensity physical activity

## Discussion

This study is the first to our knowledge to investigate longitudinal changes and tracking of total volume and accumulation patterns of physical activity and SED across three timepoints from early to late childhood among boys and girls. Given increasing evidence of the importance of patterns of physical activity and SED for health [3–5, 31] and the need to identify opportunities to intervene, these findings are timely. We found that total volume and bouts of SED increased for both sexes across the three time points, while total volume and bouts of LPA decreased. Although we found a small decrease in total volume of MPA for girls only, time spent in ≥ 1-min bouts of MPA increased and total volume of VPA increased for both sexes. Our findings are consistent with previous studies that have shown small increases in physical activity across 1–2 year periods in children aged 3–4 years at baseline [32–34]. Similarly, although Taylor et al. [35] observed a decline in MVPA from age 3 to 4 years, they found that it remained relatively consistent from age 4 to 7 years. They concluded that the increases in SED observed in their study predominantly occurred at the cost of activities undertaken at a light intensity.

Findings from this study highlight the need for interventions from an early age to focus on reducing SED by shifting children’s movement along the activity spectrum. The amount of time spent in SED is potentially largely driven by screen time. Screens are ubiquitous and evidence suggests that fewer than 20 % of preschool aged children meet recommendations for screen time [6]. Additionally, findings from Jones et al. [12] show that TV viewing is particularly stable during early childhood and from early to middle childhood, which may be driving the stability of overall SED. As such, a potential strategy for reducing SED may be to focus on reductions in sedentary screen time. Our findings also suggest that it is important to establish high levels of MVPA in early childhood, as children may then be more likely to maintain higher levels over time. However, young children are unlikely to be able to achieve long bouts of MVPA from a physiological or cognitive perspective and tend to have limited tolerance for sustained MVPA [36]. As such, interventions should focus on reducing SED, maximising time spent in LPA, and promoting adequate levels of total MVPA accumulated in shorter bouts, to include appropriate rest breaks.

In terms of tracking, our findings suggest that total volume of SED, LPA, MPA and VPA are moderately to highly stable from early to later childhood for both boys and girls. However, tracking of the bouts variables was less consistent. Although there was moderate to strong tracking of shorter (≥ 1- to ≥ 10-min) bouts of SED for both sexes, ≥ 15- and ≥ 20-min bouts tracked weakly. This is largely consistent with findings from Carson et al. [15], who found moderate tracking of total SED and shorter SED bouts (1–4 and 5–9 min), but no tracking of ≥ 10-min SED bouts among 3- to 5-year-olds over a 1 year period. These collective findings are likely because children accumulate SED differently as they get older, highlighted by the large increases in average time spent in longer bouts in our study.

In terms of the physical activity pattern variables, we found moderate to strong tracking of shorter bouts and weak tracking of longer bouts of LPA, and weak to moderate tracking of bouts of MPA (≥ 1-min). While VPA bouts of ≥ 1-min and ≥ 5-min tracked moderately for boys, we found weak tracking of these bouts for girls. It may be that engagement in longer and higher intensity bouts of physical activity is more variable as children age, given that they are more physically challenging to achieve. As children transition from preschool to primary school, there may also be fewer opportunities for them to engage in spontaneous physical activity. For girls specifically, it is likely that social norms increasingly influence their physical activity patterns as they age [37, 38]. The comparatively little amount of time spent in ≥ 5-min LPA, ≥ 1-min MPA, ≥ 1-min VPA and ≥ 5-min VPA bouts may also partly explain the weaker tracking than that of ≥ 1-min LPA bouts. In addition, tracking coefficients for ≥ 5-min bouts of LPA and VPA for girls were not statistically significant. This may be due to lack of power given the small numbers of children participating in bouts of this duration. For example, only around one quarter of girls included in analyses spent time in ≥ 5-min bouts of VPA at each time point.

Contrary to previous research that has found that physical activity tends to track more strongly for boys than girls [28, 39], we found that while tracking was evident for both sexes, average total time in SED and all intensities of physical activity tended to be more stable for girls than boys. This, combined with the findings that SED was consistently higher, and MPA and VPA consistently lower for girls than boys over the three time points, suggests that girls may need additional support to reduce SED and engage in sufficient levels of MPA and VPA for health benefits. This underscores the need for physical activity interventions that target girls specifically, particularly as evidence consistently shows that girls are less active than boys [40–42]. Future research should aim to determine what factors influence girls’ and boys’ activity patterns, in order to design effective interventions.

A limitation of the current study is the loss to follow up. The HAPPY Study was initially designed as a cross-sectional study, so follow-up samples were limited to those who had consented to be re-contacted. However, response rates among those contacted at both follow up time points were around 75 %. Strengths of the current study include the longitudinal design, which allowed the investigation of changes and tracking over three time points, and objective measures of sedentary time and physical activity. In addition, the examination of changes in and tracking of pattern variables is novel.

## Conclusions

Children’s activity patterns changed over time from preschool (3–5 years) to late primary school (9–11 years), with total volumes and short bouts of physical activity and SED tracking more strongly than longer bouts. Although MPA and VPA appeared to increase slightly, total volume and bouts of SED increased considerably more and, of concern, seemed to replace LPA. Future research should aim to determine what factors influence these changes in activity patterns so that effective interventions can be designed to ensure that children establish and maintain healthy physical activity and SED levels from a young age.

## Data Availability

The datasets analysed for the current study are not publicly available due to ethical restrictions related to the consent given by participants at the time of study commencement. An ethically compliant dataset may be made available by the corresponding author on reasonable request and upon approval by the Deakin University Human Research Ethics Committee.

## Abbreviations

LPA: Light-intensity physical activity
MPA: Moderate-intensity physical activity
MVPA: Moderate- to vigorous-intensity physical activity
PA: Physical activity
SED: Sedentary time
VPA: Vigorous-intensity physical activity
